# Improvement in the Blood Urea Nitrogen and Serum Creatinine Using New Cultivation of *Cordyceps militaris*

**DOI:** 10.1155/2022/4321298

**Published:** 2022-03-23

**Authors:** Chih-Hui Yang, Wen-Shuo Kuo, Jun-Sheng Wang, Yi-Ping Hsiang, Yu-Mei Lin, Yi-Ting Wang, Fan-Hsuan Tsai, Chun-Ting Lee, Jiun-Hua Chou, Huei-Ya Chang, Lung-Shuo Wang, Shu-Chi Wang, Keng-Shiang Huang

**Affiliations:** ^1^Deptarment of Biological Science and Technology, I-Shou University, Kaohsiung, Taiwan; ^2^Taiwan Instrument Research Institute, National Applied Research Laboratories, Hsinchu, Taiwan; ^3^Pharmacy Department of E-Da Hospital, Kaohsiung, Taiwan; ^4^School of Chemistry and Materials Science, Nanjing University of Information Science and Technology, Nanjing, China; ^5^Department of Biotechnology and Chemical Engineering, I-Shou University, Kaohsiung, Taiwan; ^6^School of Chinese Medicine for Post-Baccalaureate, I-Shou University, Kaohsiung, Taiwan; ^7^Amulette Chinese Medicine Clinic, Taipei, Taiwan; ^8^Department of Chinese Medicine, Sin-Lau Hospital, Tainan, Taiwan; ^9^School of Medicine for International Students, I-Shou University, Kaohsiung, Taiwan

## Abstract

**Background:**

Chronic kidney disease (CKD) is a critical public health issue with a huge financial burden for both patients and society worldwide. Unfortunately, there are currently no efficacious therapies to prevent or delay the progression of end-stage renal disease (ESRD). Traditional Chinese medicine practices have shown that *Cordyceps militaris* (*C. militaris*) mycelia have a variety of pharmacologically useful properties, including antitumor, immunomodulation, and hepatoprotection. However, the effect of mycelial *C. militaris* on CKD remains unclear.

**Methods:**

Here, we investigated the effects of *C. militaris* mycelia on mice with CKD using four types of media: HKS, HKS with vitamin A (HKS + A), CM, and CM with vitamin A (CM + A).

**Results:**

The results at day 10 revealed that the levels of blood urea nitrogen (BUN) were significantly lower in the HKS (41%), HKS + A (41%), and CM + A (34%) groups compared with those in the corresponding control groups (nephrectomic mice). The level of serum creatinine in the HKS + A group decreased by 35% at day 10, whereas the levels in the HKS and CM + A groups decreased only by 14% and 13%, respectively, on day 30. Taken together, this is the first report using four new media (HKS, HKS + A, CM, and CM + A medium) for *C. militaris* mycelia. Each medium of mycelial *C. militaris* on CKD exhibits specific effect on BUN, serum creatinine, body weight, total protein, and uric acid.

**Conclusions:**

Taken together, this is the first report using four new media (HKS, HKS + A, CM, and CM + A medium) for *C. militaris* mycelia. Each medium of mycelial *C. militaris* on CKD exhibits specific effects on BUN, serum creatinine, body weight, total protein, and uric acid. We concluded that treatment with *C. militaris* mycelia cultured in HKS or CM + A medium could potentially prevent the deterioration of kidney function in mice with CKD.

## 1. Introduction

Chronic kidney disease (CKD) is a prevalent global health problem [1]. CKD is a general term for heterogeneous disorders affecting kidney structure and function [2]. Patients with CKD have an increased risk of end-stage renal disease (ESRD) [3, 4]. Existing pharmacological agents focus on the complications related to CKD treatments, such as hyperlipidemia, diabetes, and hypertension rather than specifically treating CKD itself [5–8], making the study of renal protection an emerging medical science. Previous studies have focused on homeodomain-interacting protein kinase 2 (HIPK2) because it is a transcriptional regulator of gene expression involved in tubular injury and fibrosis [9, 10]; however, specific HIPK2 inhibitors are not commercial available. In addition, blocking the renin-angiotensin-aldosterone system has been shown to reduce both the risk of hyperkalemia progression and the recurrence rate [11, 12]. Although the renin-inhibiting drug aliskiren has previously been administered alongside angiotensin-converting enzyme inhibitors or angiotensin receptor blockers regularly, it is now being used more conservatively because of its severe side effects [13].

It has been reported that the risk factors for chronic kidney disease were age, race, obesity, diabetes, low birth weight, high blood pressure, and family history [14]. The risk of CKD morbidity and mortality remains considerably high, with CKD patients usually receiving renal replacement therapy, such as dialysis and kidney transplantation. Thus, novel treatments must be developed. In recent years, herbal therapies have provided an alternative treatment option for CKD [15, 16]. In addition, many researches exhibited that proper Chinese herbal medicine prescriptions have a positive effect on CKD, which can significantly reduce the risk of ESRD in patients with CKD and improve the long-term survival rate of patients with CKD [17]. For example, the efficacy of several herbs, including *Radix Astragali*, *Rheum officinale*, *Panax ginseng*, and *Lycopus lucidus,* on kidney diseases has been investigated [18–21]. Some herbs have shown promising results in decreasing proteinuria or increasing serum albumin. Others, however, contain toxic ingredients, such as aristolochic acid or heavy metals, which may adversely affect kidney function and induce nephropathy [22]. Although they have been used frequently in some developing countries, reports of their efficacy remain controversial. Therefore, developing an efficacious compound derived from a natural product for treating CKD is an urgent concern.

*Cordyceps* belongs to a fungus family and is a type of traditional Chinese medicine in which parasitic insect larvae grow and gradually turn into a mature fruiting body. *Cordyceps sinensis* (*C. sinensis*) and *Cordyceps militaris* (*C. militaris*) are two well-known *Cordyceps* species. For many years, *C. sinensis* was used to treat fatigue, renal and pulmonary dysfunction, hyperglycemia, hyperlipidemia, and arrhythmia [23], and its effects in CKD patients and kidney transplant recipients have been studied [24–26]. Generally, *C. militaris* is relatively amenable to mass production [27] and exhibits both diverse and specific pharmacological properties [28–30]. In addition, a previous study conducted by our group investigated the anticancer functions of *C. militaris* and demonstrated that its mycelial fermentation could regulate the mitogen-activated protein kinases (MAPK) signal pathway to halt the cell cycle, stimulate chromosomal DNA breakdown, and ultimately cause both apoptotic and autophagic death of cultured glioblastoma cells [31]. Therefore, *C. militaris* would be employed for its renoprotective effects in this study.

Mycelial *C. militaris* was recently developed as a popular functional food in Asia. Extracts from the *C. militaris* fruiting body could significantly delay the progression of renal malfunction induced by subtotal nephrectomy [32]. Studies on the effect of *C. militaris* mycelia on CKD, however, are rare. This study will test our hypothesis that mycelial *C. militaris* can potentially prevent the deterioration of kidney function in mice with CKD. Four types of media will be designed and employed to incubate mycelial *C. militaris*: (i) HKS, (ii) HKS + A, (iii) CM, and (iv) CM + A. For 30 days, mice in the treatment groups will receive daily administration by oral gavage of *C. militaris* mycelia cultured in one of the four different media, whereas the mice in the sham control (C) and nephrectomic control (Nx) groups will receive distilled water. These tests are expected to clarify the safety of mycelial *C. militaris* as a functional food.

## 2. Materials and Methods

### 2.1. Materials

*C. militaris* mycelia (BCRC 32219) were purchased from the Bioresource Collection and Research Center at the Food Industry Research and Development Institute (Hsinchu, Taiwan). Glucose was purchased from J. T. Baker (EU). Malt extract, peptone, and yeast extract were purchased from Becton Dickinson (Franklin Lakes, NJ, USA). Vitamin A was purchased from Sigma-Aldrich (St. Louis, MO, USA) and agar was purchased from High Standard Enterprise Co., Ltd. (Taiwan), respectively. Biochemical assay kits for kidney function detection were obtained from Arkray (Kyoto, Japan).

### 2.2. Fungus Media Preparation

Mycelial *C. militaris* was incubated in four types of media, namely CM, CM + A, HKS, and HKS + A. The CM medium contained 2% malt extract, 2% agar, 0.1% peptone, and 2% glucose [33]. The HKS medium was a modification of a medium devised by Prof. Huang Keng-Shiang and contained 2% malt extract, 2% agar, 0.2% peptone, and 0.2% yeast extract. The CM and HKS media differed in the concentrations of the peptone and yeast extract. The HKS + A and CM + A media each contained an additional 1% vitamin A in the base medium. A 0.5 × 0.5 cm square of *C. militaris* mycelium was cut and transplanted onto each plate and incubated at 25°C. After the *C. militaris* was cultured in the different media for 30 days, the fungal mycelia were carefully scraped off the surface of the solid medium using a knife. The collected fungal mycelia powders were freeze-dried (EYELA FDU-1100) in vacuum at −54°C for 48 hours. The freeze-dried *C. militaris* mycelia powders were stored at −20°C until use.

### 2.3. CKD Mice Model Construction

The five-sixth nephrectomy was the most established chronic procedure that mimics progressive renal failure after the loss of renal mass. The CKD mice were established after a two-step, five-sixth nephrectomy as described previously [34, 35]. Briefly, the left kidney was exposed and cut at the upper and lower one-third poles. The 2/3 of the extrarenal branches of the renal artery of the left kidney was ligated and then followed by a completely right nephrectomy after one week. Animals were returned to their individual cages postsurgery for at least two weeks before uremia was induced. The procedures were approved by the Institutional Animal Care and Use Committee of I-Shou University (Approval No: IACUC-ISU-101025).

### 2.4. Experimental Procedure

Freeze-dried powders of *C. militaris* mycelia were soaked in distilled water (10 mg/mL) at room temperature. Two weeks after the second nephrectomy, the mice were randomly divided into treatment groups (*n* = 4 to 6 per group) and treated with one of the four types of the *C. militaris* mycelia solutions, incubated in HKS, HKS + A, CM, or CM + A medium, by oral gavage for 30 consecutive days. Sham group and the Nx mice were administered the equivalent volume of distilled water without *C. militaris* mycelia. Body weight was measured and blood samples were collected on days 1, 10, and 30, respectively. At the end of the treatment period, the mice were sacrificed using CO_2_. The kidneys were dissected and washed with phosphate-buffered saline and placed in 10% neutral buffered formalin for subsequent histological processing. Blood samples were collected from the periorbital venous sinus. Blood urea nitrogen (BUN), serum creatinine, total protein, and uric acid were measured using commercially available kits using an automated biochemical analyzer (SPOTCHEM EZ SP-4430) according to the manufacturer's instructions (Arkary, Inc., Kyoto, Japan).

### 2.5. Animal Feedings

The Institute of Cancer Research male mice (approximately 30 g) were supplied by BioLASCO Taiwan Co., Ltd. and kept in standard cages at a constant temperature of 22 ± 1°C with a 12-hour light-dark cycle. Animals were fed with regular mouse chow and tap water ad libitum. The animals used in this study were housed and cared for in accordance with the NIH Guide for the Care and Use of Laboratory Animals.

### 2.6. Biochemical Analysis of Blood Samples

Blood samples were collected from the periorbital venous sinus. Plasma samples were centrifuged at 12,000 rpm for 10 min at 4°C and stored at −20°C before analysis. Blood urea nitrogen (BUN), serum creatinine, total protein, and uric acid were measured using commercially available kits using an automated biochemical analyzer (SPOTCHEM EZ SP-4430) according to the manufacturer's instructions (Arkary, Inc., Kyoto, Japan).

### 2.7. Renal Histological Analysis

The mice kidneys were embedded in paraffin blocks, dissected into 3-*μ*m-thick sections, and processed with Harry's hematoxylin-eosin (HE) stain following the standard procedure. The renal glomeruli and tubules were examined and photographed for future analysis. Images were captured using a color video camera (VKC150, Hitachi, Tokyo, Japan) connected to a microscope (DP72, Olympus, Center Valley, PA, USA) and blindly analyzed by an experienced pathologist. The mean glomerular cross-sectional area was obtained by calculating the mean area of approximately 8 to 15 individual glomeruli using the program Image J.

### 2.8. Statistical Analysis

All data are presented as mean ± standard error of the mean (*n* = 4 to 6 per group). Data were analyzed using two-way analysis of variance followed by Bonferroni post hoc tests (SigmaPlot version 10.0, San Jose, CA, USA). *p* < 0.05 was considered statistically significant.

## 3. Results

**Figure 1:**
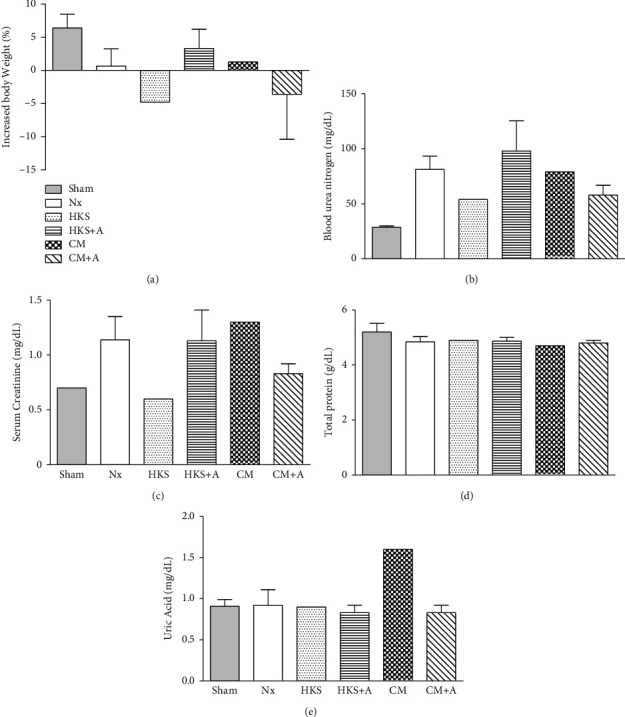
Body weight and biochemical plasma values in healthy (sham) and uremic mice, with and without *C. militaris* mycelia treatment at day 30. (a) The influence of increased body weight. Percent change in body weight was calculated using the equation (body weight of day 30–body weight of day 1)/body weight of day 1) × 100%; (b) the influence of BUN; (c) the influence of serum creatinine; (d) the influence of total protein; (e) the influence of uric acid. Partial nephrectomy ICR mice (weighing approximately 30 g each) were divided into four groups and treated with *C. militaris* mycelia (3 mg dried mycelia dissolved in 0.3 mL water) cultured in HKS, HKS + A, CM, or CM + A medium by oral gavage for 28 days, whereas the Nx control group received distilled water. Data were collected at the end of the experiment. All data are expressed as mean ± standard error of the mean.

**Figure 2:**
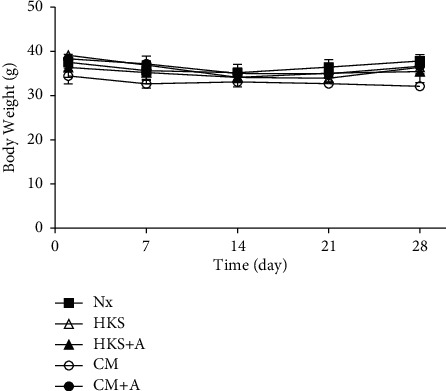
Body weight growth curves from uremic mice, with and without *C. militaris* mycelia treatment. Partial nephrectomy ICR mice (weighing approximately 30 g each) were divided into four groups and treated with *C. militaris* mycelia (3 mg dried mycelia dissolved in 0.3 mL water) cultured in HKS, HKS + A, CM, or CM + A medium by oral gavage for 28 days, whereas the Nx control group received distilled water. Body weight measurements were taken every day after treatment with *C. militaris* mycelia water solution or distilled water until day 30. Values are expressed as mean ± standard error of the mean.

The control and nephrectomy mice were treated with and without *C. militaris* mycelia, once a day by oral gavage for 30 days. The sample number in each treatment was 4–6 mice per group. The percent increase in body weight and the values of blood urea nitrogen (BUN), serum creatinine, total protein, and uric acid at day 30 are summarized in Figure 1. At the end of the experiments, the percent increase in body weight was lower in uremic mice who underwent a partial nephrectomy (Nx, 0.66 ± 2.58%) than in the control mice (Sham, 6.41 ± 2.92%), revealing that uremic mice experienced growth retardation during the experimental period. Body weights from the uremic mice with and without *C. militaris* treatments are shown in Figures 1(a) and 2. The results indicated that the body weight in HKS, HKS + A, or CM + A group was decreased compared to that of the Nx group at day 30 (Figure 1(a)). However, none of the *C. militaris*-treated groups exhibited obvious differences in body weight compared with the uremic control mice during the experimental period. These data in Figure 2 suggest that the treatment of *C. militaris* mycelia did not significantly affect the weight of the uremic mice.

**Figure 3:**
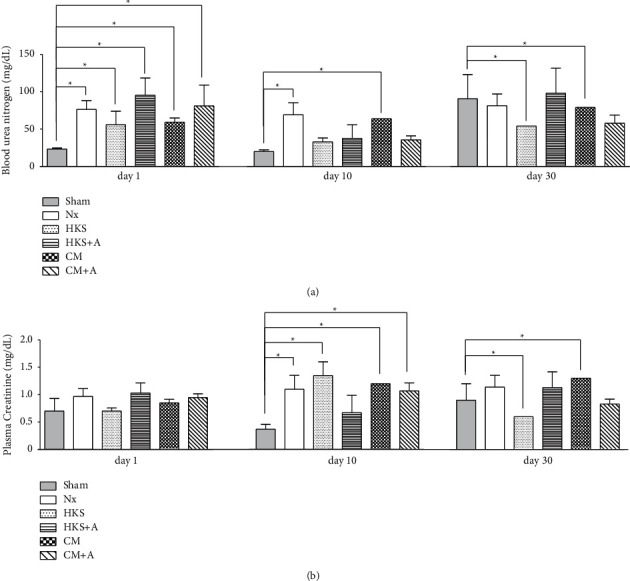
Relative levels of (a) BUN and (b) serum creatinine in the uremic mice with or without *C. militaris* mycelia treatment at days 1, 10, and 30. Partial nephrectomy ICR mice (weighing approximately 30 g each) were divided into four groups and treated with *C. militaris* mycelia (3 mg dried mycelia dissolved in 0.3 mL water) cultured in HKS, HKS + A, CM, or CM + A medium by oral gavage for 28 days, whereas the Nx control group received distilled water. Values are expressed as the mean ± standard error of the mean.

Plasma biochemical values are presented in Figures 1(b), 1(c), and 3. As expected, the BUN and serum creatinine were significantly higher (2.83-fold and 1.63-fold, respectively) in the uremic mice than in the Nx control mice, demonstrating that this uremic mouse model precisely reflects progressive CKD (Figures 1(b) and 1(c)). The levels of BUN had a pattern of decreasing on day 10 in uremic mice fed with *C. militaris* mycelia cultured in HKS (41%), HKS + A (41%), and CM + A (34%) media compared with the uremic control mice (Nx) (Figure 3(a)). The level of serum creatinine was reduced on day 10 in the HKS + A (35%) group and on day 30 in the HKS (14%) and CM + A (13%) groups (Figure 3(b)). The level of serum creatinine increased in the HKS (93%) and CM (41–53%) groups on day 10 (Figure 3(b)). Differences in body weight, total protein, and uric acid were not detected between the sham and Nx mice (data not shown). In addition, the total protein levels were similar in different groups indicating that *C. militaris* treatment from different cultivations could prevent the progression of CKD (Figure 1(d)). The uric acid also had similar levels, except for the CM group, which had higher uric acid levels (Figure 1(e)).

**Figure 4:**
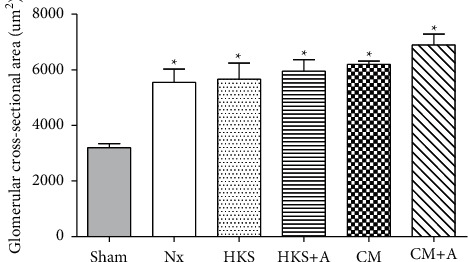
Renal glomerular cross-sectional areas in the sham and uremic mice with or without *C. militaris* mycelia treatment. Four groups of partial nephrectomy ICR mice (weighing approximately 30 g) received a daily administration of media-treated *C. militaris* mycelia water solution (3 mg dried mycelia dissolved in 0.3 mL water) from culture in HKS, HKS + A, CM, or CM + A medium by oral gavage for 30 days, whereas the sham and Nx control groups received distilled water. The uremic groups showed significant hypertrophy of the remnant glomeruli compared with the sham group. Values are expressed as mean ± standard error of the mean.

**Figure 5:**
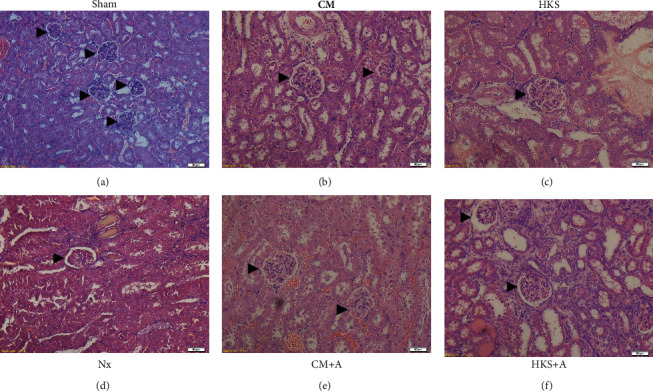
Light micrographs of the kidney glomerular and renal tubular sections stained with hematoxylin–eosin (HE × 200). (a) Sham control mice; (b) uremic control mice (Nx); uremic mice treated with *C. militaris* mycelia cultured in (c) CM, (d) CM + A, (e) HKS, or (f) HKS + A medium. Enlarged glomerular and proteinaceous casts within the tubular lumen were observed in the Nx, CM, CM + A, and HKS groups (b-e) but not in HKS + A medium group f. a-f scale bars were 50 *μ*m.

To compare the effects of different *C. militaris* mycelia-treated groups on the kidney, histological data from the healthy and uremic mice were examined.  Figure 4 shows the renal glomerular cross-sectional areas in the sham and uremic mice with or without *C. militaris* mycelia treatments. The mean glomerular cross-sectional area was obtained by calculating the mean area of approximately 8 to 15 individual glomeruli using the program Image J. There are no statistically significances between the uremic groups. In the sham control group, renal tissue sections exhibited normal morphology (Figure 5(a)), whereas the renal tissue sections from the uremic mice exhibited enlarged glomeruli and dilated renal tubules surrounded by flat epithelial cells (Figures 5(b)–5(f)). The glomerular cross-sectional area was significantly larger in the uremic mice (1.74-fold) than in the healthy (sham) mice. An irregular arrangement of tubules and colloid casts in the lumen of the tubules was also present in the injured kidney. The enlargement of the remnant glomeruli demonstrates that the remnant kidney in this uremic mice model may be the result of long-lasting high hemodynamic pressure and the overloading of metabolic waste.

## 4. Discussion

Recently, artificial cultures of *C. militaris* mycelia are a popular research topic [22–27]. For example, researchers have investigated different media to artificially culture *C. militaris* mycelia, such as silkworm pupa, solid rice medium, germinated soybean medium, and soybean broth [34–39]. Many studies have investigated different concentrations of carbon and nitrogen in the media to explore the best conditions for the growth of *C. militaris* mycelia [40–45]. Previous studies have shown that *C. militaris* mycelia contain specific pharmacologically active components, such as cordycepin, polysaccharides, ergosterol, and mannitol, and that they can be used effectively for various medicinal purposes or as functional foods [46–48]. Differences in the structural characterizations, immunomodulation of polysaccharides, and the antioxidant activity of *C. militaris* mycelia grown on different media have also been explored [49]. The current study focused on the renoprotectant properties of *C. militaris* mycelia *in vivo* and compared the effects of *C. militaris* mycelia incubated in new media. The results exhibited that mice with kidney injury treated with *C. militaris* mycelia cultivated on HKS or CM + A medium had signs of improvement in their kidney function. The HKS medium contains yeast extract and two-fold more peptone than the CM medium; moreover, it has obvious glucose scarcity. The HKS medium is a high-nitrogen medium, whereas the CM medium is a high-carbon medium. We are the pioneer investigators to use HKS and CM media for the artificial culturing of *C. militaris* mycelia in order to understand the differential effects that a high-nitrogen medium (HKS) and a high-carbon medium (CM) have on the biosynthetic changes of *C. militaris* mycelia.

Vitamin A is a group of fat-soluble organic substances that include retinol, retinal, retinoic acid, several pro-vitamin A carotenoids, and *beta*-carotene [50]. Carotenoids, which are vitamin A precursors, are isoprenoid molecules synthesized *de novo* by photosynthetic plants, fungi, and algae and are responsible for the orange, yellow, and (some of the) red colors of various fruits and vegetables. Vitamin A and its related derivatives have been shown to inhibit several biological functions, including tumor growth, angiogenesis, metastasis, and cell proliferation, as well as induce cell apoptosis and differentiation [51, 52]. The presence of vitamin A in the medium has stimulated the biosynthesis of *β*-carotene in *Phycomyces blakesleeanus* [53], suggesting that vitamin A is a strong activator of carotenogenesis in *Phycomyces*. As shown in Figure 3(b), on day 30 in the CM + A group, *C. militaris* mycelia started to exert an inhibitory effect on serum creatinine. By contrast, treatment with *C. militaris* mycelia in the CM group (without vitamin A) had no inhibitory effect on serum creatinine levels. In addition, the inhibitory effect was not observed in *C. militaris* mycelia when vitamin A was added in the KHS medium. Therefore, the function of vitamin A in fungi is unclear. We suspect that vitamin A may provide the material for *C. militaris* mycelia to synthesize secondary metabolites and thereby affect cell apoptosis. The role of vitamins in media should be further studied to support the industrial production of *C. militaris* mycelia [54].

Dong et al. exhibited the effect of *C. militaris* mycelia on BUN and serum creatinine levels in streptozotocin-induced diabetic rats [55]. The inhibition effects on BUN, creatinine, uric acid, and protein revealed the protection of *C. militaris* extracts against diabetic nephropathy. They presumed that the *C. militaris* extract has great potential for diabetes treatment. Yu et al. investigated the renal injury-reducing effect of *Cordyceps militaris* treatment in type 2 diabetic nephropathy mice [56]. They reported that blood glucose, renal dysfunction markers (*e.g.,* serum creatinine and kidney-to-body weight ratio), and pathological alterations in renal tissues were significantly mitigated and ameliorated after treatment. Herein, we demonstrated that the levels of BUN in uremic mice were inhibited by *C. militaris* mycelia cultured in the HKS, HKS + A, or CM + A medium after 10 days, and in the HKS or CM + A medium after 30 days (Figure 3(a)). In addition, we demonstrated that the levels of serum creatinine were inhibited by *C. militaris* mycelia cultured in the HKS + A medium after 10 days, and in the HKS or CM + A medium after 30 days (Figure 3(b)). We found that *C. militaris* mycelia powders harvested from the HKS and CM + A media have a stronger effect on decreasing the BUN and serum creatinine levels to remedy kidney injury in CKD mice. *C. militaris* mycelia harvested from different media may exert different compounds that have various protective effects in CKD. Taken together, treatment with *C. militaris* mycelia may improve the biochemistry index of CKD, but it could not repair the damages in this uremic mice model.

Khan et al. exploited emulgel with aspirin for topical application [57]; this concept could be further applied in our study. We propose that *C. militaris* mycelia can be loaded in micro/nanobeads for controlled drug release applications. In addition, Fattepur et al. and Zharif et al. use the ethanolic extract of plants to study the toxicological, pharmacological activity, or synergistic effect with antibiotics [58, 59]. The bioactive compounds' extraction was a good idea for our further study. Therefore, we propose that the ethanolic extraction of *C. militaris* mycelia would be used and studied in the future.

We propose that future studies would be focused on the detailed molecular mechanism of immunomodulation activity, the histopathological changes in kidney tissues, and the new formulation of *C. militaris* mycelia.

## 5. Conclusion

We have successfully observed the renoprotective effects of *C. militaris* mycelia in new media on mice with CKD. Kidney surgery was performed on young mice (approximately 30 g) to induce uremia. This is the first report using four new media (HKS, HKS + A, CM and CM + A medium) for *C. militaris* mycelia. Each medium of mycelial *C. militaris* on CKD exhibits a specific effect on the BUN, serum creatinine, body weight, total protein, and uric acid. The results revealed that the HKS medium of mycelial *C. militaris* has the strongest effect on decreasing the BUN and serum creatinine levels to remedy kidney injury in CKD mice. The HKS + A medium of mycelial *C. militaris* was effective on 10th day, but not suitable for long-term use (e.g., 30 days). The CM medium of mycelial *C. militaris* was not an expert in inhibiting the effects on BUN or serum creatinine. The CM + A medium of mycelial *C. militaris* has an acceptable effect on uremic mice. We presume that *C. militaris* mycelia cultivated in the HKS or CM + A medium has the potential to be a new functional food for CKD.

## Data Availability

Data supporting the results of our study are included in the manuscript.

## Abbreviations

BCRC: Bioresource Collection and Research Center
BUN: Blood urea nitrogen
C: Control mice
CKD: Chronic kidney disease
*C. militaris*: *Cordyceps militaris*
*C. sinensis*: *Cordyceps sinensis*
ESRD: End-stage renal disease
HE: Hematoxylin-eosin
HIPK2: Homeodomain-interacting protein kinase 2
ICR: Institute of Cancer Research
MAPK: Mitogen-activated protein kinases
NBF: Neutral buffered formalin
Nx: Nephrectomic control mice
PBS: Phosphate-buffered saline.
